# Emergency and Critical Care Medicine: An Essential Component of All Specialties and Practices

**DOI:** 10.3389/fvets.2017.00165

**Published:** 2017-10-11

**Authors:** Karol Ann Mathews

**Affiliations:** ^1^University of Guelph, Guelph, ON, Canada

**Keywords:** veterinary medicine, critical care, emergency treatment, gut microbiome, pain management

Veterinary emergency and critical care medicine is one of the fastest growing specialties in veterinary medicine. Likened to two specialties joined as one, in a continuum of care and in a partnership working with other specialties, veterinary emergency and critical care focuses on the immediate needs of a severely ill or injured animal and also on management of the critical medical and surgical patient beyond the primary problem. A wide spectrum of illnesses, injuries, and toxicities ranging from acute kidney injury to snakebite and from severe trauma to diabetic ketoacidosis and hyperlipidemia, are experienced by dogs, cats, horses, and other veterinary patients throughout the world. Board-certified members of the American and European Colleges of Veterinary Emergency & Critical Care are termed “criticalists” because they provide immediate, essential, and intensive care and management for these animals. In addition to board-certified emergency clinicians working in academia and in private practice, primary care veterinarians also provide emergency medical care at the front line in both specialty and non-specialty veterinary practices. Basic and clinical research in veterinary emergency and critical care medicine that is accessible to all veterinarians is essential to the ongoing advancement and development of the field.

Veterinary emergency and critical care medicine encompasses all organ systems and associated functions, anatomical structures, physiology, and pathophysiology, yet the criticalist manages the patient as a whole. Similarly, although other specialists typically manage a specific surgical or medical problem, criticalists frequently manage multiple comorbidities. As such, emergency and critical care clinicians face an important challenge: they must achieve a high level of broad interdisciplinary expertise while also requiring deep knowledge in the core areas of the specialty. Core areas of emergency and critical care medicine include pain management, mechanical/positive pressure ventilation, transfusion medicine, coagulation disorders, fluid and colloidal therapy, CPR and cardiorespiratory disorders, sepsis and antimicrobial use, trauma management, and acute plant and chemical toxicities. Management of critical illness and injury requires continual assessment, interpretation, and management of the patient’s status, including vital signs, and acid–base, electrolyte, hematologic, cardiovascular, respiratory, renal, neurological, gastrointestinal, and nutritional status. Laboratory technical skills, point-of-care testing, and ultrasonographic skills are frequently required for evaluation of the emergent and critically ill patient. The veterinary criticalist has the expertise to balance these layers of knowledge and to prioritize attention to medical and surgical issues requiring immediate intervention.

In this Specialty Grand Challenge, I will highlight some of the core areas in the field of veterinary emergency and critical care medicine, in particular, those newly emerging or where additional research is especially needed to improve the quality of medicine and critical care. I also will emphasize the need for interdisciplinary research that is mutually beneficial between the field of veterinary emergency and critical care and other veterinary specialties, and the importance of knowledge and educational exchange among emergency and critical care specialists and veterinary practitioners.

## Pain Management

Most veterinary patients experience pain associated with the primary problem or with the various procedures they undergo during hospitalization. The degree of pain can range from mild to excruciating, requiring single or multimodal therapy with on-going assessment and adjustment. Studies evaluating the pharmacologic (1, 2) and non-pharmacologic aspects of analgesic regimens (3) to eliminate pain are an essential contribution to patient management for a wide range of injuries and illness. A study led by Indiana University School of Medicine researchers demonstrates how electroacupuncture triggers a neurological mechanism that can help promote tissue repair and relieve injury-induced pain (4). Other benefits of acupuncture have also been studied in human medicine (5), an area welcoming veterinary studies. Other studies reporting on integrative techniques that enhance recovery and reduce pain would also be an important contribution to this section of Frontiers (6). Analgesia, in addition to sedation and local or general anesthesia, is required to facilitate diagnostic imaging procedures and to manage the primary or secondary problems these patients experience. Critical care patients are fragile and benefit from carefully selected and performed protocols that help ensure detailed and consistent care (7). Research studies highlighting best practices in pain management are vital to reduce morbidity and mortality in veterinary emergency and critical care patients, especially in areas where little information currently exists (8).

## The Antimicrobial Challenge

Many veterinary patients require antibiotics for their primary problem, and others are susceptible to infection. Antibiotic stewardship is thus a very important area for the criticalist, especially with the current concerns for global antimicrobial resistance. Studies following the pattern of resistance in the community and in veterinary practices are an important contribution to managing the ill or injured patient (9–11). The gut microbiome is impacted by critical illness either alone or with antibiotic usage, changing from a commensal and synergistic relationship to one of microbial virulence. This alteration in the behavior and population of the gut microbiome has the potential to propagate a pathologic host response leading to multiple organ dysfunction syndrome (12–15). The challenge for veterinary clinicians and investigators is identifying the necessity for, and appropriate selection of, antibiotic therapy, keeping in mind the need for a healthy gut microbiome and concurrent nutritional support. Numerous strategies to manipulate the microbiome have been used with varying success in critically ill human patients (16); it is essential to validate these strategies for use in the critically ill animal.

## Interdisciplinary, Clinical, and Translational Research in Emergency and Critical Care

Research studies conducted by other veterinary specialties are an integral part of understanding and managing animals presented for emergencies and requiring critical and continual care. While the diagnostic and/or specific medical and surgical management of the emergent and critically ill patient may be similar to that of the otherwise healthy patient, an added requirement is consideration of comorbidities that preclude a routine approach to patient management. Research in humans has contributed significantly to our understanding of critical care in animals; similarly, much of the research intended to improve treatment of human trauma, blood loss, and wound management have relied on animal models for evaluation and testing. Translational research relevant to both animals and humans is also essential in advancing the field. I feel the approach would be that areas in common with human and veterinary medicine can be given careful consideration for veterinary patients, keeping in mind the physiological and other potential differences. A careful, selective approach to the transfer of information can be beneficial when associated with the clinical assessment, study and publication. Finally, clinical research is an important aspect that continues to advance emergency and critical care through evidence based medicine. Numerous areas of emergency and critical care clinical research including a careful, selective approach to the transfer of information can be beneficial when associated with the clinical assessment, study and publication ng prognostic indicators (Sofa Q scores, animal trauma triage, shock index, biomarkers, etc.), point of care diagnostic tests (FAST ultrasound, lactate, indirect blood pressure, etc.), and sepsis, SIRS and pain scoring, to name just a few, have helped advance standards of care and improve patient management (17, 18).

## Optimizing Emergency Medicine in Primary Care Practice

An important challenge in our field is the limited availability of emergency and critical care specialists in many areas of the country and of the world, requiring primary emergency treatment by a non-specialist. For example, eye injuries and dental or oral injuries occur in horses and small animals and require immediate attention and management, even where specialty equipment and knowledge may not be available. A focus on triage and the initial management of these and other complex emergencies would be of great benefit to animal patients and the emergency veterinarians who must handle them. Recent discussions have centered on whether to implement training programs for veterinarians, outside of the current board-certification process, that focus on emergency medicine and surgery knowledge and skills for general practitioners. Such training programs would better equip the front-line emergency veterinarian with the skills to effectively manage these patients. Educational tools and articles focused on relevant issues for practitioners who engage in emergency medicine are also much needed. Finally, assessment and validation of teaching methodologies and the standards required to become proficient in specific skills is an exciting research area that is currently receiving attention in veterinary emergency and critical care. Recent articles have demonstrated training courses can improve technical proficiency in emergency cardiac ultrasound performed by non-cardiologists (19, 20), and further studies in this and related research fields are likely to influence ECC training guidelines and requirements in the future.

## Conclusion

The specialty of veterinary emergency and critical care medicine has made enormous strides in advancing progress in core areas and, by extension, in all specialties. Procedural invasiveness and patient comorbidities are no longer obstacles to patient survival when initial and continuing critical care is provided by veterinarians with specialty training in emergency and critical care. The specialty section of Veterinary Emergency and Critical Care Medicine in *Frontiers in Veterinary Science* will help address these challenges by providing a unique and optimal venue for the publication of clinical and basic research in the field. The section’s close alliance with other specialty sections in the journal and with other Frontiers journals will help facilitate trans- and interdisciplinary collaboration and awareness. In addition, because Veterinary Emergency and Critical Care Medicine is open-access, its articles are available to everyone, from board-certified specialists to academicians, to translational research scientists, and to the primary care clinicians who practice emergency medicine at the front line. Clinicians and scientists are invited to submit their clinical and experimental research studies, evidence-based recommendations, reviews, technical reports, and commentaries to the Veterinary Emergency and Critical Care Medicine section of *Frontiers in Veterinary Science*. The availability of high quality information in the field will be available for global impact and interactive peer review and will effectively reach primary care veterinary practitioners as well as specialists.

## Author Contributions

The author confirms being the sole contributor of this work and approved it for publication.

## Conflict of Interest Statement

The author declares that the research was conducted in the absence of any commercial or financial relationships that could be construed as a potential conflict of interest. The reviewer KM and handling editor declared their shared affiliation.
